# Temperature-mediated invocation of the vacuum state for switchable ultrawide-angle and broadband deflection

**DOI:** 10.1038/s41598-018-32977-z

**Published:** 2018-10-09

**Authors:** Andriy E. Serebryannikov, Akhlesh Lakhtakia, Majid Aalizadeh, Ekmel Ozbay, Guy A. E. Vandenbosch

**Affiliations:** 1 ESAT-TELEMIC, Katholieke Universiteit Leuven, 3000 Leuven, Belgium; 2 Faculty of Physics, Adam Mickiewicz University, 61-614 Poznań, Poland; 3 NanoMM–Nanoengineered Metamaterials Group, Department of Engineering Science and Mechanics, Pennsylvania State University, University Park, Pennsylvania 16802 USA; 4 Material Architecture Centre and Department of Electronics Engineering, Indian Institute of Technology (BHU), Varanasi, Uttar Pradesh 221005, India; 5 Nanotechnology Research Center (NANOTAM), Bilkent University, 06800 Ankara, Turkey; 6 Department of Physics, Department of Electrical Engineering, and National Institute of Materials Science and Nanotechnology (UNAM), Bilkent University, 06800 Ankara, Turkey

## Abstract

Temperature-mediated appearance and disappearance of a deflection grating in a diffracting structure is possible by employing InSb as the grating material. InSb transits from the dielectric state to the plasmonic state in the terahertz regime as the temperature increases, this transition being reversible. An intermediate state is the vacuum state in which the real part of the relative permittivity of InSb equals unity while the imaginary part is much smaller. Then the grating virtually disappears, deflection being impossible as only specular reflection can occur. This ON/OFF switching of deflection and relevant angular filtering are realizable over wide ranges of frequency and incidence angle by a temperature change of as low as 20 K. The vacuum state of InSb invoked for ON/OFF switching of deflection and relevant angular filtering can also be obtained for thermally tunable materials other than InSb as well as by using non-thermal mechanisms.

## Introduction

Terahertz, mid-infrared, and near-infrared devices that are tunable in real time have been attracting considerable attention for about a decade. Different dynamic tuning mechanisms have been investigated. They include electric^1–3^, magnetic^4,5^, thermal^6–10^, and photonic^11^ control of electromagnetic constitutive parameters. Infiltration by gaseous species is also a tuning mechanism^12^. Although a dramatic change in the electromagnetic state of a material is strictly unnecessary for dynamic tunability^1,4,7^, the transition of a material from/to the dielectric state to/from the plasmonic state affords exciting possibilities in various tuning scenarios. The epsilon-near-zero state (relative permittivity = 0), an intermediate state between the plasmonic and dielectric ones, has garnered attention as well^2,13–16^.

In this paper, we introduce the *vacuum state* (relative permittivity = 1) as an intermediate state to enable and control wideband and wide-angle deflection by a diffraction grating. If a material is suddenly made to function as vacuum, it is as if that material has vanished in thin air! That effect lies at the heart of our work reported here.

For the purpose of demonstration, we restrict our theoretical consideration to the effect of nearly perfect deflection (blaze) in the reflection mode, in very wide ranges of the frequency and the incidence angle^17,18^, these ranges being wider than those realizable from most of other known approaches to angular filtering^19–21^. The geometry of the chosen structure is similar to that suggested in ref.^18^, except for the significant modification that the grating in the studied structure is made of InSb, a thermally tunable material. Thermal control of carrier concentration in InSb allows us to tune the grating from the plasmonic/dielectric state to the dielectric/plasmonic state^6,9^. Most importantly, the grating *virtually vanishes* in the intermediate vacuum state. Thus, the grating can be made to either appear or disappear, depending on the absolute temperature *T*. In a manner of speaking, the appearance or disappearance can be considered to be a change in shape, the intermediate vacuum state being tantamount to infinite compression of the grating to a flat object of zero thickness. Thus, the proposed mechanism can be considered as an alternative to a recently suggested one that employs shape-memory polymers to change the shape of the corrugations of a grating^22^. While InSb is usable in this way only in the terahertz spectral regime and its physical dimensions do not change, shape-memory polymers can serve very well in a very wide spectral regime but undergo actual mechanical deformation. Additionally, we note that the materials in which the vacuum state is exhibited as a result of the plasmonic-to-dielectric state transition, also exhibit an epsilon-near-zero intermediate state. Thus, an epsilon-near-zero grating can also be manifested in our structure, albeit at another temperature; however, that issue lies beyond the scope of this paper.

In the remainder of this paper, first we briefly present the temperature-dependent relative permittivity of InSb in the terahertz regime, and then we present numerical results to demonstrate the temperature-mediated appearance and disappearance of the wideband and wide-angle deflection grating.

## Geometry and Constitutive Properties

**Figure 1:**
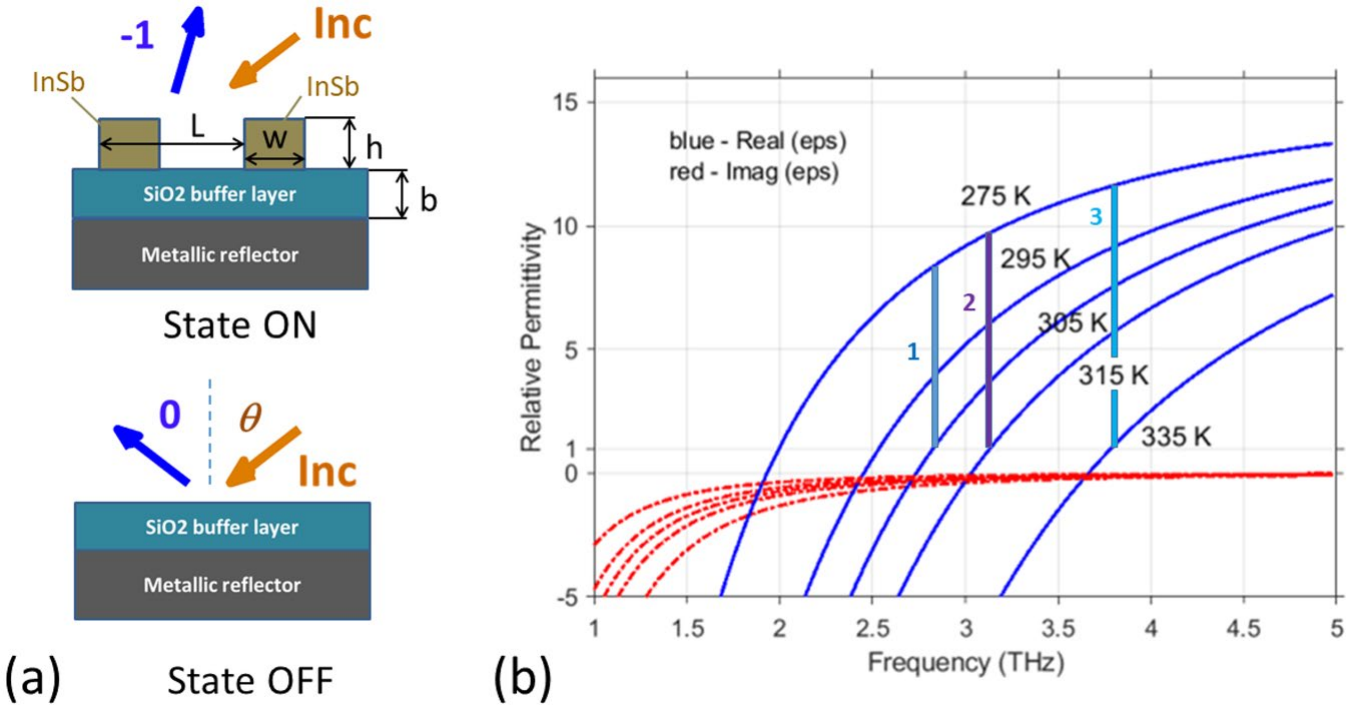
(**a**) Schematics in which the deflection grating is present in the ON state but effectively disappears in the OFF state; arrows labeled Inc, 0, and −1 show the propagation directions of the incident plane wave, the specularly reflected Floquet harmonic of order *m* = 0, and the nonspecularly reflected, i.e., deflected Floquet harmonic of order *m* = −1, respectively, in the ideal case. (**b**) Relative permittivity of InSb at different temperatures. The blue solid lines are for the real part and the red dash-dotted lines are for the imaginary part. Thick vertical lines (identified by 1, 2, 3) present three examples of transition from the vacuum state to the dielectric state by decreasing *T*.

The geometry of the chosen structure is shown in the upper panel of Fig. 1(a). The structure comprises a lamellar grating composed of rods having rectangular cross-section of height *h* and width *w*, which is placed on the top of a silicon-dioxide buffer layer of thickness *b* and backed by a metallic reflector. The grating height *h* and period *L* are chosen so that the reflection of a linearly polarized incident plane wave as a Floquet harmonic of order −1 is close to perfect in wide frequency and incidence-angle ranges. If this deflection grating is made of InSb, then it is almost *electromagnetically indistinguishable* from the vacuum above it at a certain temperature, resulting in only specular reflection, as shown in the lower panel of Fig. 1(a).

Two structures are considered for illustration: the period *L* = 94 *μ*m for the grating in structure A and *L* = 62.7 *μ*m for the grating in structure B. We fixed *h* = 0.2*L*, *b* = 0.1385*L*, and *w* = *h* for both structures A and B. Thus, structure B is a geometrically downscaled version of structure A. However, the structures are not mutually scalable in an electromagnetic sense^23,24^, because InSb is a dispersive material, so that its relative permittivity at a fixed temperature is frequency dependent as shown in Fig. 1(b). Hence, both structures A and B should exhibit different diffraction properties despite their geometric relationship.

At terahertz frequencies, the relative permittivity of InSb is described by the Drude model^6–8^

1 $${\varepsilon }_{InSb}(\omega )={\varepsilon }_{\infty }-\frac{{\omega }_{p}^{2}}{{\omega }^{2}-i\gamma \omega }.$$

Here, the exp (*iωt*) dependence on time *t* is implicit with *ω* as the angular frequency and $i=\sqrt{-\,1}$; *ε*_∞_ = 15.68 is the high-frequency relative permittivity; ${\omega }_{p}=\sqrt{N{q}_{e}^{2}/{\varepsilon }_{0}{m}^{\ast }}$ is the plasma angular frequency, with *q*_*e*_ = −1.6 × 10^−19^ C, *ε*_0_ = 8.854 × 10^−12^ F m^−1^, and m^*^ = 1.3665 × 10^−32^ kg being the electron charge, free-space permittivity, and effective free-carrier mass, respectively; and *f* = *ω/2π* is the frequency. Finally, *γ* = 0.1*π* THz is the damping constant^7,25,26^. For the temperature range from 160 K to 350 K, the intrinsic career density *N* (in m^−3^) is given by^26–28^

2 $$N=5.67\times {10}^{20}{T}^{\mathrm{3/2}}\,\exp \,(-\,\frac{0.13}{{k}_{B}T}),$$

where *k*_*B*_ = 8.62 × 10^−5^ eV K^−1^ is the Boltzmann constant.

Figure 1(b) presents Re[*ε*_*InSb*_] and Im[*ε*_*InSb*_] vs *f* for selected values of *T*. At a fixed frequency in the chosen spectral regime, InSb undergoes a transition from the dielectric state (Re[*ε*_*InSb*_] > 0 at lower *T*) to the plasmonic state (Re[*ε*_*InSb*_] < 0 at higher *T*). The frequency at which Re[*ε*_*InSb*_] = 1 (and |Im[*ε*_*InSb*_]| is substantially smaller than Re[*ε*_*InSb*_]) shifts from 2 THz at 275 K to 3.78 THz at 335 K. Therefore, one should use the frequency corresponding to Re[*ε*_*InSb*_] = 1 at the higher possible temperature, in order to obtain a high-permittivity contrast from vacuum when Re[*ε*_*InSb*_] > 1. Then, a transition from the dielectric state to the vacuum state becomes available by increasing *T*. In turn, the dielectric state can be achieved from the vacuum state by an appropriate decrease of *T*. As is shown later, the main operating regime that enables ON/OFF switching is based on this transition. Note that in order to obtain a transition from the plasmonic state to the vacuum state, the temperature has to be lowered.

## Results and Discussion

Suppose that the chosen structure is illuminated by a linearly polarized plane wave incident at arbitrary angle *θ*; see Fig. 1(a). Let the electric field be oriented parallel to the grating lines (i.e., rods), so that the incident plane wave is *s* polarized. The reflected field is a superposition of Floquet harmonics of order *m*, |*m*| ≥ 0. We calculated the reflection coefficients *r*_*m*_ by using the technique described in the Methods section. The reflectance *R*_*m*_ ∝ |*r*_*m*_|^2^ is either finite and lies between 0 and 1, or it is identically zero, depending on whether the *m*th-order Floquet harmonic is propagating or evanescent.

**Figure 2:**
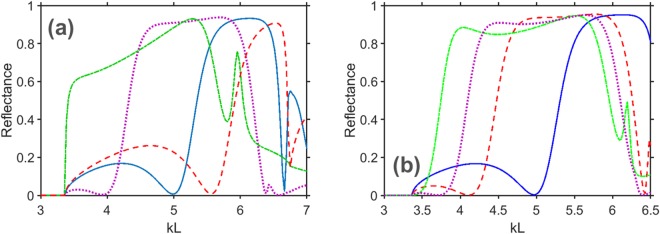
(**a**) Nonspecular reflectance *R*_−1_ vs. *kL* of structure A at *T* = 305 K (dashed red line), 295 K (solid blue line), 275 K (dotted violet line), and 245 K (dash-dotted green line). (**b**) Nonspecular reflectance *R*_−1_ vs. *kL* of structure B at *T* = 335 K (solid blue line), 315 K (dashed red line), 305 K (dotted violet line), and 295 K (solid green line). Calculations were made for *θ* = 60°.

Figure 2 presents the nonspecular reflectance *R*_−1_ as a function of *kL* for both structures A and B at several values of *T*, *k* being the free-space wavenumber. Deflection into the *m* = −1 order at a fixed *f* can be switched ON or OFF by simply changing *T*. For example, *R*_−1_ ≈ 0.01 at *T* = 335 K but *R*_−1_ > 0.9 at *T* = 305 K for structure B when *kL* = 4.97; see Fig. 2(b). The switching is similar for structures A and B, although it is obtained in different ranges of temperature. Hence, the structure can be geometrically re-scaled at the design stage to achieve tunability in a particular range of temperature. This re-scalability is a general feature that is not restricted to the two chosen structures.

The key difference between the ON and OFF states of the structure is connected to the characteristic electromagnetic properties of InSb. In the plasmonic state, we have a metallic grating which typically creates a bell-shaped frequency dependence of *R*_−1_, e.g., in the vicinity of *kL* = 4.5 at *T* = 305 K for structure A in Fig. 2(a). In the vacuum state (Re[*ε*_*InSb*_] = 1), the grating disappears, although its effect still lingers like the grin of the Cheshire cat^29^ due to non-zero Im[*ε*_*InSb*_]. In the dielectric state, Re[*ε*_*InSb*_] reaches values larger than 5, yielding very high *R*_−1_. Besides, *R*_−1_ may become weakly dependent on frequency, e.g., at *kL* ∈ (4.8, 5.9) and *T* = 315 K in Fig. 2(b). The thermally controllable transition from Re[*ε*_*InSb*_] = 1 to Re[*ε*_*InSb*_] > 5 is essentially responsible for the dynamic tunability of the chosen structures. Numerical results demonstrating the possible effects of variations in *h* and *w* are presented in Supplementary Information. They show that even with 10% deviation from the selected value of *h* and 16.7% deviation from the selected value of *w*, all the features in the variations of *R*_−1_ vs. *f* and *T*, which are observed in Fig. 2 and needed for ON/OFF switching, do remain.

Two more features observed in Fig. 2 should be noticed. As an example of the first of them, switching in Fig. 2(a) between *R*_−1_ ≈ 0 and *R*_−1_ > 0.9 is achieved at *kL* = 5 by decreasing *T* from 295 K to 275 K, corresponding to the transition from the vacuum state to the dielectric state. However, we also observe the opposite case at *kL* = 6.4, when *R*_−1_ ≈ 0.01 for 275 K and *R*_−1_ = 0.9 for 295 K. This case has nothing to do with the vacuum state, being realized in the dielectric state fully due to the suppression of diffraction and anomalous absorption. As a result, we may obtain dual-band ON/OFF switching, i.e., co-existing ON/OFF and OFF/ON switching regimes in two distinct frequency ranges located quite close to each other. Clearly, 275 K and 295 K is not a unique pair of *T*-values, at which this advanced switching functionality can be obtained.

The second feature is partially seen in Fig. 2(b) at *kL* = 4.97. The vacuum state is achieved at 335 K, as mentioned previously. In the dielectric state, *R*_−1_ > 0.88 is *immune* to temperature variations in a wide range, at least between 245 K (not shown) and 315 K.

**Figure 3:**
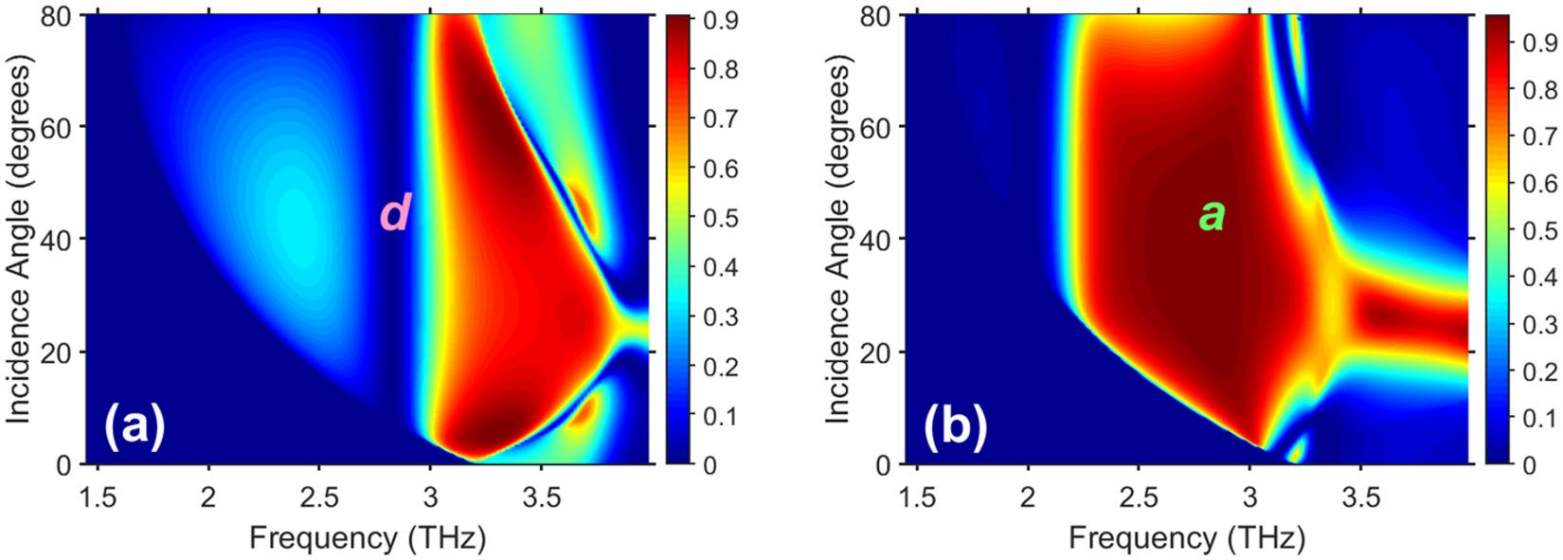
Nonspecular reflectance *R*_−1_ of structure A in the (*f*, *θ*)-plane at (**a**) *T* = 305 K and (**b**) *T* = 275 K. The labels *a* and *d* indicate the regions in which the grating appears and effectively disappears, respectively.

Figure 3 presents *R*_−1_ of structure A as a function of *f* and *θ* for two values of *T*. Large regions in the (*f*, *θ*)-plane have *R*_−1_ > 0.8, so that strong deflection via nonspecular reflection is a general feature of the chosen structure, not merely an incidental result of fortuitous choices of *f* and *θ*.

By lowering *T* from 305 K to 275 K in the vicinity of *f* = 2.83 THz, one can achieve the scenario in which the grating effectively disappears at the higher temperature (Re[*ε*_*InSb*_] = 1) but deflects strongly at the lower temperature (Re[*ε*_*InSb*_] > 1), as is clear on comparing Fig. 3(a) with Fig. 3(b). This happens in a wide *θ*-range, which extends at *f* = 2.83 THz from ${\theta }_{th}^{(-\,1)}={7}^{^\circ }$, the threshold for *R*_−1_ to become nonzero, to about 75°, at which *R*_−1_ starts to decrease and *R*_0_ starts to increase towards unity as *θ* → 90°. Hence, efficient wide-angle ON/OFF switching of deflection and relevant angular filtering is possible. In line with Lorentz reciprocity, the range of directions of the deflected wave should coincide with the range of *θ* in which *R*_−1_ is significant, and that range includes the backscattering case.

Let us note that other approaches to deflection typically need more complex structures than the studied ones, e.g., gradient structures with subwavelength elements^30,31^, including metasurfaces^32^. Moreover, it is expected to be quite challenging to obtain highly efficient, ultrawide-angle, and broadband deflection in such structures, even in static configurations. The same remains true for the structures based on the classical echelette gratings.

**Figure 4:**
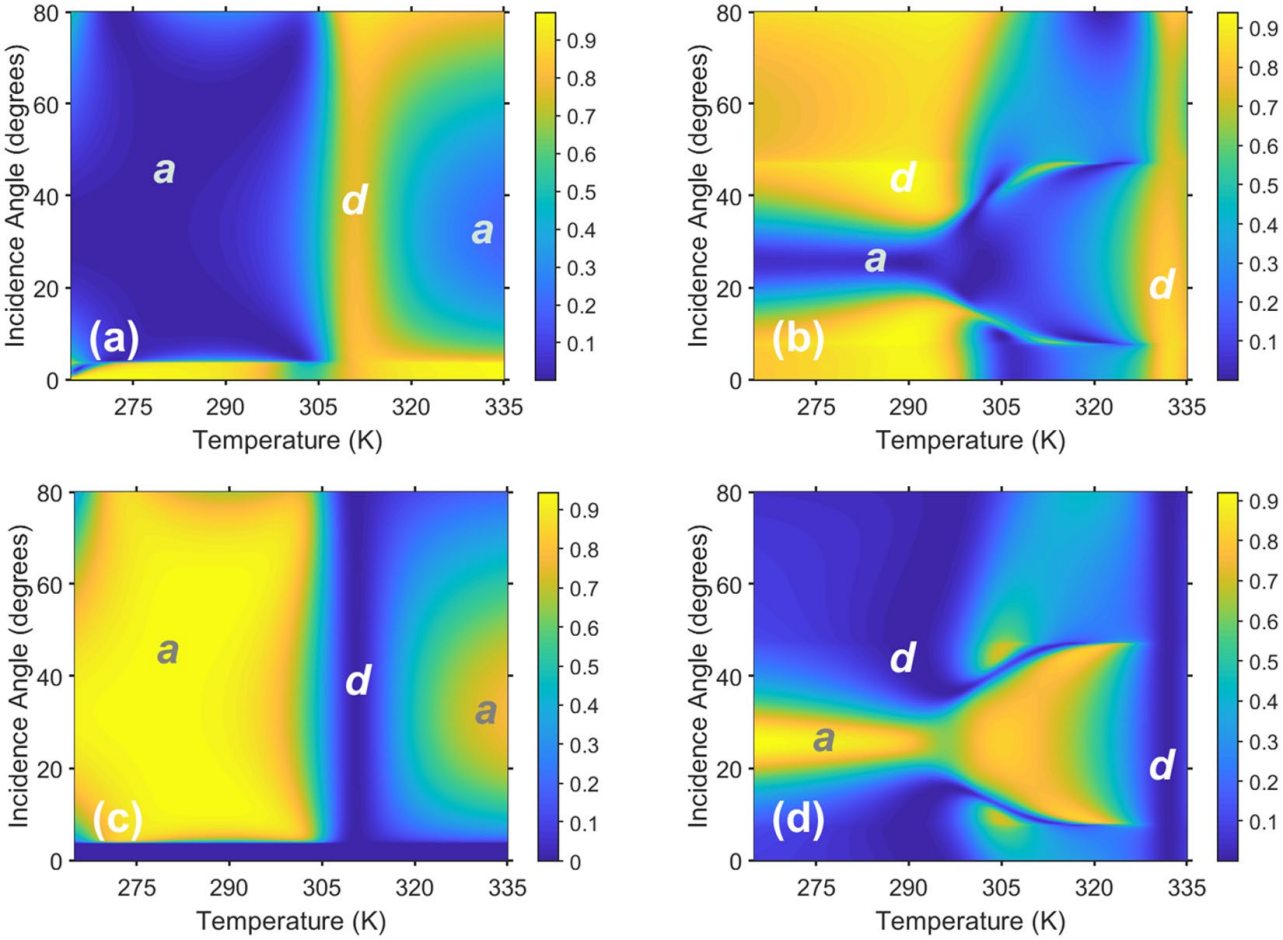
(**a**,**b**) Specular reflectance *R*_0_ and (**c**,**d**) nonspecular reflectance *R*_−1_ of structure A in the (*T*, *θ*)-plane at (**a**,**c**) *f* = 2.995 THz (*kL* = 5.896) and (**b**,**d**) *f* = 3.658 THz (*kL* = 7.255). The labels *a* and *d* indicate the regions in which the grating appears and effectively disappears.

Next, let us consider both *R*_0_ and *R*_−1_ of structure A as functions of *T* and *θ*, for two selected values of *f* in Fig. 4. Although somewhat unusual, this way of presenting results provides further insight into the proposed approach. One can see that effect of the same variations in *T* and/or *θ* can be different, depending on the operating frequency. In Fig. 4(a,c), the propagation threshold ${\theta }_{th}^{(-\,\mathrm{1)}}$ = 3.5°. In Fig. 4(d), the order *m* = −1 propagates at any *θ*. In Fig. 4(c), a very large region exists in which *R*_−1_ > 0.9. In fact, strong deflection is immune to the variations in *T* and *θ* within this region. Note that this immunity occurs in the high-permittivity dielectric state of InSb, similarly to one of the features in Fig. 2 discussed heretofore. The largest values of *R*_−1_ are obtained in the vicinity of *T* = 280 K. For *T* > 290 K, Re[*ε*_*InSb*_] varies rapidly with *T* and its real part is equal to unity near *T* = 310 K. Here, the grating tends to virtually disappear and *R*_0_ rises. A further increase of *T* leads to the transition of InSb to the plasmonic state, so that the grating diffracts significantly for *T* > 320 K. The results presented in Figs. 4(a,c) clearly show how *T* should be chosen for ON/OFF and OFF/ON switching, as well as for gradual transition from the ON state to the OFF state and vice versa. Only a 30-K difference in *T* is required. Cooling from an initial *T* = 310 K makes the grating appear. Warming from an initial *T* = 280 K leads the grating to virtually vanish at *T* = 310 K.

Comparing Figs. 4(a,c) with Figs. 4(b,d), one can see how the change in frequency may affect the shape and location of the region of strong deflection in the (*T*, *θ*)-plane. Some of the diffraction orders with |*m*| > 1 may also be of the propagating type and contribute to the energy-balance equation, as well as significant absorption may occur, so that the wide-angle dominant behavior of *R*_−1_ is not guaranteed. Generally, the shape and size of the regions of large *R*_−1_ are mainly determined in both (*f*, *θ*) and (*T*, *θ*) planes by the common effect of high-order propagation threshold and the specifics of the variation of Re[*ε*_*InSb*_] in the quiescent state (i.e., the operating point).

**Figure 5:**
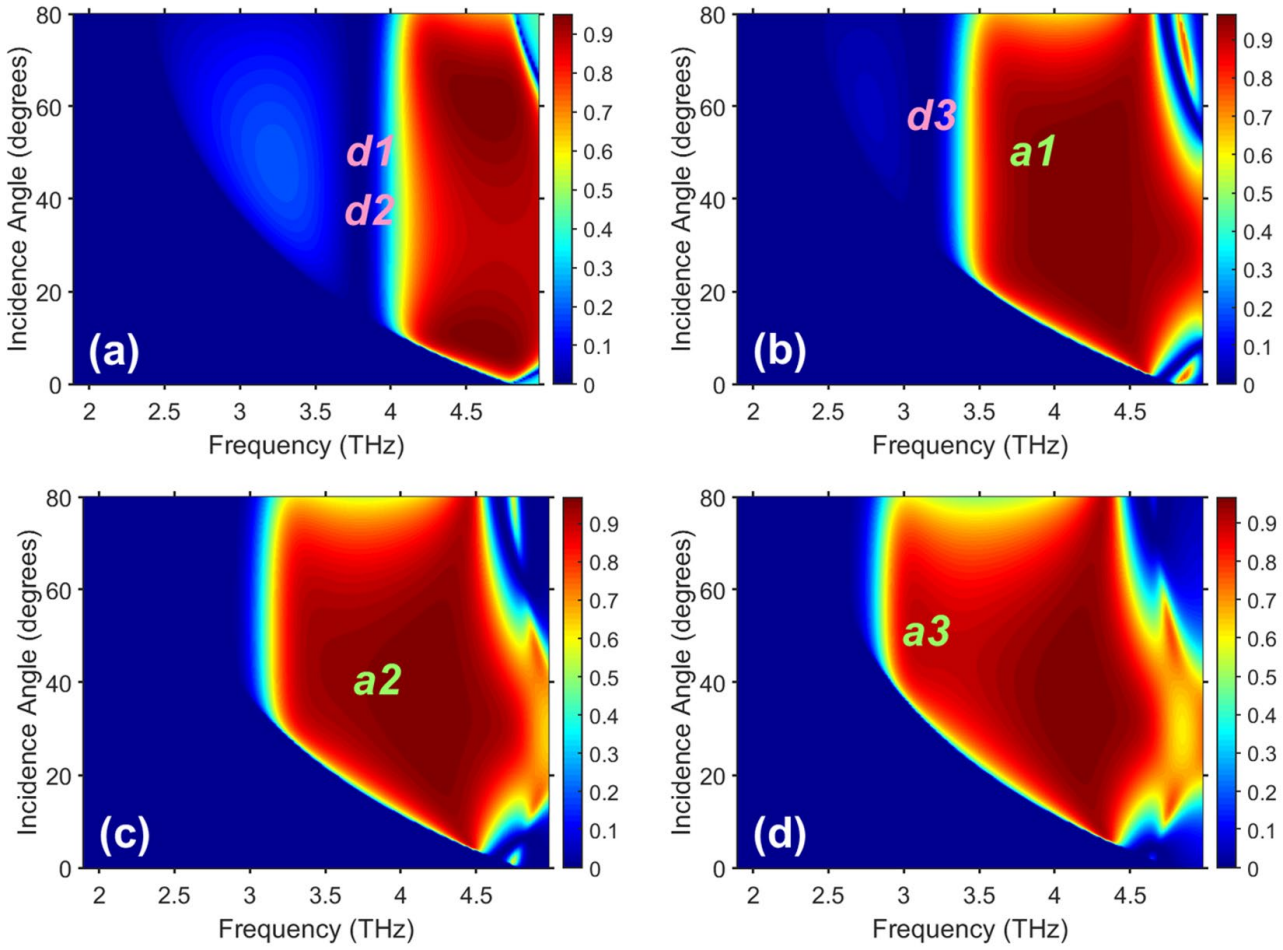
Nonspecular reflectance *R*_−1_ of structure B in the (*f*, *θ*)-plane at (**a**) *T* = 335 K, (**b**) *T* = 315 K, (**c**) *T* = 305 K, and (**d**) *T* = 295 K. The labels *an* and *dn*, *n* ∈ {1, 2, 3}, indicate the regions in which the grating appears and effectively disappears, respectively.

An important practical feature is that the grating can be re-designed to deliver ON/OFF switching at higher temperatures than in Figs. 3 and 4. As an example, Fig. 5 shows *R*_−1_ of structure B as a function of *f* and *θ* at four values of *T*. Large regions in the (*f*, *θ*)-plane have *R*_−1_ > 0.8, these regions being larger in Figs. 5(c,d) than the ones in Fig. 3. Just a 20-K difference in *T* is sufficient to obtain ON/OFF switching between the regions *a*1 and *d*1 in Figs. 5(a,b) or between regions *a*3 and *d*3 in Figs. 5(b,d). However, a 30-K difference in *T* is needed to obtain ON/OFF switching between the regions *a*2 and *d*2 in Figs. 5(a,c), just the same as noted for Fig. 3. Similarly to Fig. 3, both ON/OFF switching and gradual tuning can be achieved in wide *θ*–ranges. Immunity to temperature variations at rather large values of Re[*ε*_*InSb*_] also remains.

**Figure 6:**
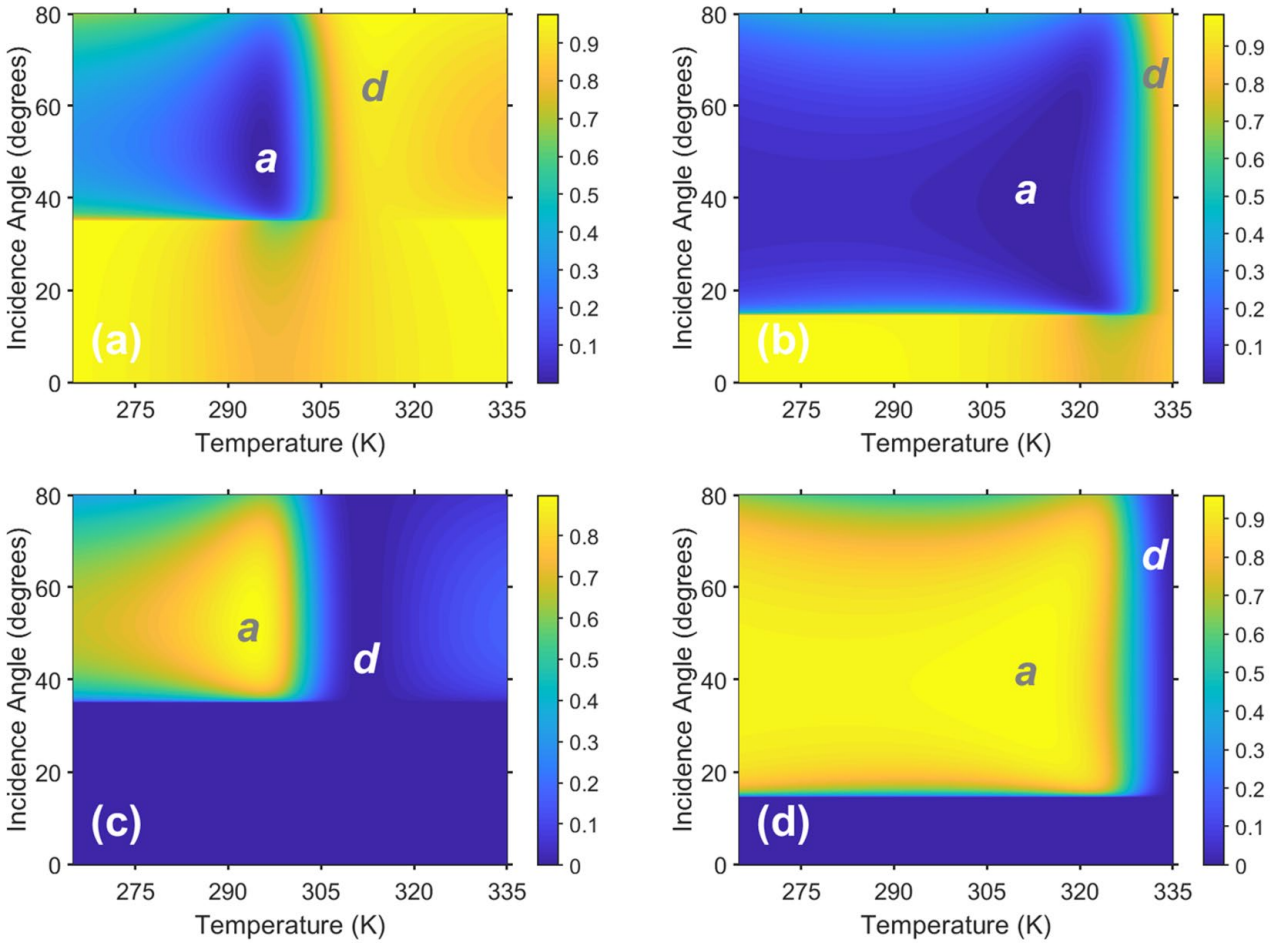
(**a**,**b**) Specular reflectance *R*_0_ and (**c**,**d**) nonspecular reflectance *R*_−1_ of structure B in the (*T*, *θ*)-plane at (**a**,**c**) *f* = 3.04 THz (*kL* = 3.99) and (**b**,**d**) *f* = 3.82 THz (*kL* = 5.014). The labels *a* and *d* indicate the regions in which the grating appears and effectively disappears.

Figure 6 shows both *R*_0_ and *R*_−1_ of structure B as functions of *T* and *θ* for two values of *f*. These values of *f* were chosen so that the Floquet harmonic of order *m* = −1 is the only nonspecular harmonic that is not evanescent, and ${\theta }_{th}^{(-\,\mathrm{1)}}$ = 35.5° in Figs. 6(a,c) and 15° in Figs. 6(b,d). The region of high *R*_−1_ is substantially expanded for *f* = 3.82 THz, as compared to that for *f* = 3.04 THz. Furthermore, the grating virtually disappears in the vicinity of *T* = 315 K in Fig. 6(c) at the lower frequency, but in the vicinity of *T* = 335 K in Fig. 6(d) it occurs at the higher frequency.

In addition to the propagation threshold dictated by the Floquet theory, in our approach we simultaneously invoke the vacuum state whereby all nonspecular diffraction disappears because the grating becomes almost electromagnetically indistinguishable at a specific temperature from the vacuum above it. By selecting a particular temperature range, we also fix the frequency range in which the vacuum state and, therefore, the suggested switching mechanism can be realized. Knowing the values of the geometric ratios *h*/*L*, *w*/*L*, and *b*/*L* necessary for ultrawide-angle and broadband deflection, we fix the frequency to unambiguously determine the linear dimensions of the structure. Since the frequency dependence of the relative permittivity of InSb does not allow scalability from one spectral regime to another^23,24^, there is also an unambiguous relationship between the used physical sizes and the used temperature range, at a given frequency. In particular, for the structures A and B, for which the set of the three geometric ratios was kept the same, the results show that while the temperatures 275 K and 295 K can be most suitable for the structure A, they might be increased by 40 K for the structure B; compare Fig. 1(a) with Fig. 1(b). On the other hand, when the temperature range of operation is fixed, the linear dimensions of the structure have to be adjusted for the chosen spectral regime. Finally, it is worth noting that Figs. 3–6 demonstrate the high capability of the suggested structures for switchable, reflection-mode angular filtering, which can be a subject of future studies.

## Concluding Remarks

Whereas materials with dynamically controllable transition from the plasmonic state to the dielectric state (or vice versa) are attracting a lot of attention mainly due to their exhibition of the epsilon-near-zero state as an intermediate state, another intermediate state–the vacuum state–has not yet been adequately studied and used. In this report, we exploited the temperature-mediated reversible transition of InSb from the dielectric state to the plasmonic state via the vacuum state in the terahertz regime to devise a diffraction structure in which deflection and angular filtering can be turned ON or OFF. The chosen structure contains a lamellar grating made of InSb. Strong nonspecular reflection of order *m* = −1 occurs when InSb is in the dielectric state and the real part of its relative permittivity is large. However, the grating almost vanishes in the vacuum state. Although the grating exists physically, it does not function as a diffraction grating because InSb is then almost indistinguishable electromagnetically from vacuum. This phenomenon occurs in a wide range of the incidence angle. Hence, switching between an ON state (nearly perfect deflection) to an OFF state (specular reflection only) is a wide-angle effect. As little as a 20-K variation in temperature is needed. The chosen structure exemplifies future thermally reconfigurable devices exploiting the vacuum state, which may include metasurfaces and surface waveguides. The obtained results give evidence of the prospects for the use of the vacuum state in various ON/OFF switching and tuning scenarios in all parts of electromagnetic spectrum. Clearly, this state can also be obtained by using other mechanisms of dynamic control, e.g., electric^2,33^ and photonic^11^ ones.

## Methods

The coupled-integral-equations technique^34^ was used to obtain all the numerical results presented in this paper. The simulations are performed by using a custom-made MATLAB code based on the fast iterative solution of the coupled integral equations in the frequency domain by using pre-conditioning. The code has been used in the earlier studies of various periodic structures. It tests convergence to preset tolerances. Its high efficiency allows us to obtain results for continuous variations of two parameters, such as those in Figs. 3–6, at reasonable computational cost.

## Electronic supplementary material

Supplementary Information
