# Supplementary material for: Temperature-mediated invocation of the vacuum state for switchable ultrawide-angle and broadband deflection
Source: Sci Rep. 2018 Oct 9;8:15044. doi: 10.1038/s41598-018-32977-z (PMC6177457; doi:10.1038/s41598-018-32977-z)
Supplement: Supplementary file 1 — Supplementary Information [file 41598_2018_32977_MOESM1_ESM.pdf]

Temperature-mediated invocation of the vacuum state for switchable ultrawide-angle and broadband deflection

Supplementary Information

A. E. Serebryannikov, A. Lakhtakia, M. Aalizadeh, E. Ozbay, and
G. A. E. Vandenbosch

In order to check the sensitivity of the discussed effects to possible deviations of the linear dimensions of the chosen structure from the selected values, we performed simulations by varying (i) the height *h* by 10%, (ii) the width *w* by 16.7%, and (iii) both *w* and *h* by 16.7% and 10%, respectively, for both structures A and B. In all three instances, we observed only qualitative differences as compared to the results for both structures presented in the main text for the selected values of *w* and *h*.

Figure S1 presents the results for R_{-1} vs. kL at $\theta = 60$ deg when *h* in structure A is either increased or decreased by 10%. By comparing Figs. S1 with Fig. 2, one can see that the main features observed for the structure A remain. At the same time, R_{-1} is sensitive to *h* so that an optimal value of this dimension needs to be selected for best performance. For instance, the width of the spectral regime for $R_{-1} > 0.8$ at $T = 275$ K increases with *h*, as follows from a comparison of Figs. S1(a) and S1(b). Although the frequency at which $R_{-1} = 0$ for 295 K due to the vacuum state (near $kL = 5$) does not depend on *h*, the frequency at which $R_{-1} = 0$ for 275 K in the dielectric state due to diffraction effects and, probably, absorption (at $kL > 6$; the grating does not disappear electromagnetically in this case) does depend on *h*. This gives us an additional degree of freedom for design of the structures with dual-band ON/OFF switching (i.e., ON/OFF and OFF/ON switching) by using the same pair of *T* values.

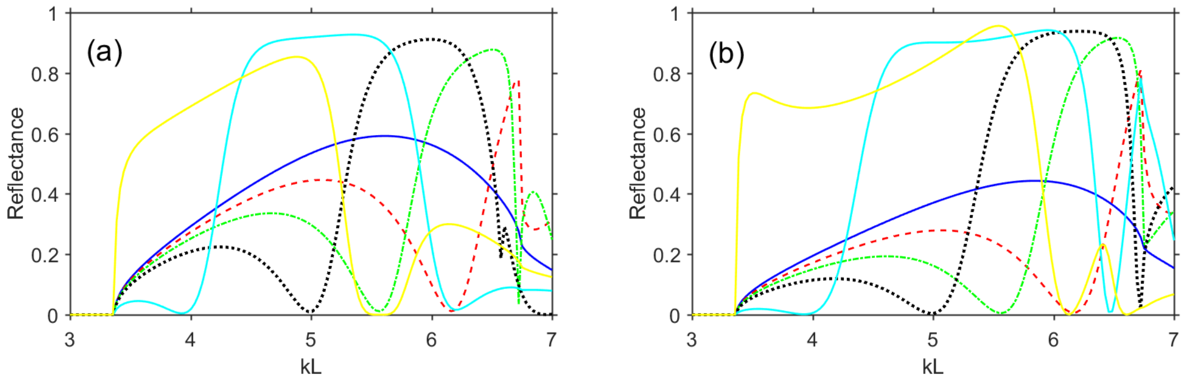

Figure S1. Nonspecular reflectance R_{-1} vs. kL at $\theta = 60$ deg for structures similar to the structure A but with *h* either (a) larger by 10% or (b) smaller by 10% than in Fig. 2. $T = 335$ K (solid blue lines), 315 K (dashed red lines), 305 K (dashed-dotted green lines), 295 K (dotted black lines), 275 K (solid cyan lines), and 245 K (solid yellow lines).

Let us note that a zero of R_{-1} due to the vacuum state may coincide with a zero of R_{-1} due to diffraction effects and absorption in the dielectric state for a specific value of kL. This happens in Fig. S1(a) near $kL = 6.2$: at 315 K (vacuum state) and 275 K (dielectric state). A similar coincidence occurs in Fig. S1(b) near $kL = 6.2$: at 315 K (vacuum state) and 245 K (dielectric case).

Figure S2 presents the results for R_{-1} vs. kL at $\theta = 60$ deg when w in structure A is either increased or decreased by 16.7 %. Finally, Fig. S3 presents the results for R_{-1} vs. kL at $\theta = 60$ deg when h and w in structure A are both either increased or decreased, by 10% and 16.7%, respectively. Again, we see that all the earlier observed features remain. This is also true regarding co-existence of the regimes of nearly-zero R_{-1} due to the vacuum state and due to diffraction and absorption.

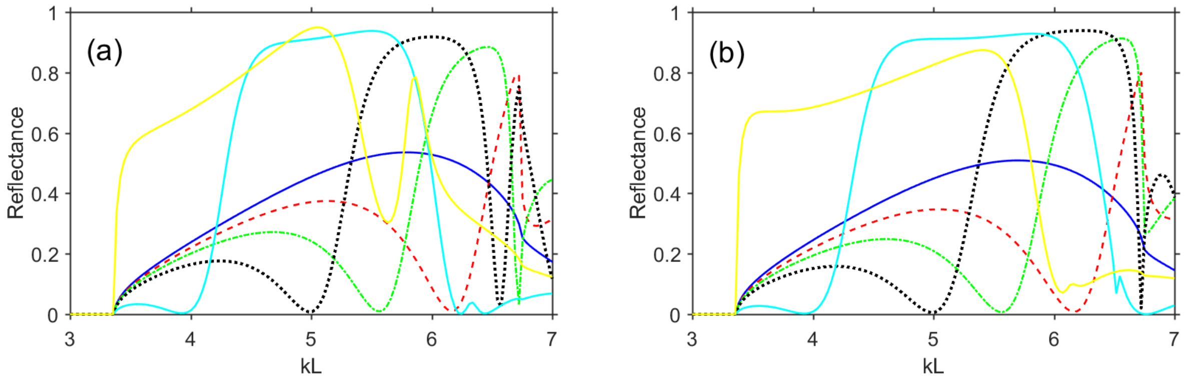

Figure S2. Nonspecular reflectance R_{-1} vs. kL at $\theta = 60$ deg for structures similar to the structure A but with w either (a) larger by 16.7% or (b) smaller by 16.7% than in Fig. 2. $T = 335$ K (solid blue lines), 315 K (dashed red lines), 305 K (dashed-dotted green lines), 295 K (dotted black lines), 275 K (solid cyan lines), and 245 K (solid yellow lines).

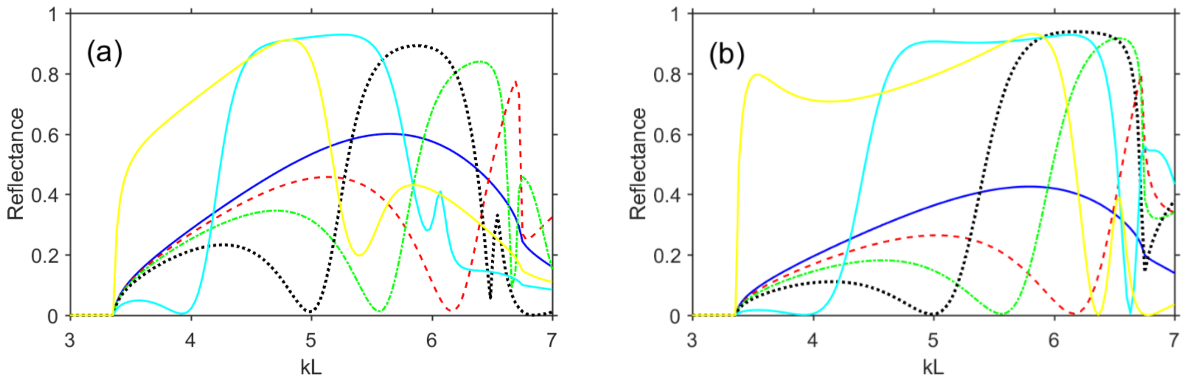

Figure S3. Nonspecular reflectance R_{-1} vs. kL at $\theta = 60$ deg for structures similar to the structure A but with (a) h larger by 10% and w larger by 16.7%, and (b) h smaller by 10% and w smaller by 16.7% than in Fig. 2. $T = 335$ K (solid blue lines), 315 K (dashed red lines), 305 K (dashed-dotted green lines), 295 K (dotted black lines), 275 K (solid cyan lines), and 245 K (solid yellow lines).

Thus, it is evident that the features needed to realize ON/OFF switching with the help of the vacuum state do not disappear when *h* and *w* are somewhat altered. The results similar to the ones in Figs. S1–S3 have also been obtained for structure B (not shown), and the conclusions we made are the same as for structure A.
